# Simultaneous small and large bowel obstruction as a consequence of internal hernia: A case report

**DOI:** 10.1016/j.ijscr.2019.02.023

**Published:** 2019-02-27

**Authors:** Malek A. Al- Omari, Mohammad A. Al-doud

**Affiliations:** Department of General Surgery, Jordanian Royal Medical Services (JRMS), Amman, Jordan

**Keywords:** Internal hernia, Intestinal obstruction, Congenital trans-mesenteric hernia

## Abstract

**Introduction:**

Intestinal obstruction ascribed to internal hernia is quite rare, especially in adults. There are no differentiating features in the presentation of intestinal obstruction due to internal hernia as compared to other causes. Delay in the diagnosis of this condition carries a considerable risk especially in a virgin abdomen. We report a rare case of internal hernia which presented as acute small and large bowel obstruction.

**Presented case:**

We report a 47- year- old male with generalized abdominal pain associated with vomiting and obstipation. The patient was in hypovolemic shock that only had a transient response to resuscitation. CT scans of the abdomen with contrast was done and showed both large and small bowel obstruction. Exploration laparotomy was done and revealed a concurrent nonviable portion of ileum and twisted sigmoid colon (volvulus) which protruded through a congenital transmesentric defect. Resection was mandatory, and repair of the defect was done.

**Discussion:**

Incidence of internal hernia generally does not exceed 1%. The diagnosis of congenital internal hernia relies on absence history of trauma, inflammatory process and abdominal surgery. Protrusion of simultaneous small and large bowels together through transmesenteric congenital gate is uncommon.

**Conclusion:**

Whether the patient presenting with intestinal obstruction has a history of undergoing previous surgeries (for any reason) or not, the diagnosis of internal hernia must be kept in mind. Coexisting involvement of both small and large bowels that need resection poses the question of the need for restoration of bowel continuity with either colostomy or ileostomy.

## Highlights

• There are no cardinal symptoms for internal hernia.
• Internal hernia must be kept as a differential diagnosis in the case of intestinal obstruction in both operated and non-operated abdomen.
• Early diagnosis both clinically and radiologically may prevent undesirable complications.
• Both patient status and surgeon's experience are essential to form the best surgical decision.
• Fine handling of bowel, assessment of viability, closure of defects and inspecting for other potential ones, and argumenting stoma formation are the main principles of surgery.

## 1 Introduction

Internal hernia is defined as protrusion of intestines through a congenital or acquired an intra-abdominal defect [1]. The presentation can be acute or chronic but there are no hallmark symptoms or signs that precisely distinguish internal hernia from other causes of intestinal obstruction [2]. In hemodynamically stable patients, early diagnosis using CT scans with IV contrast as a modality of imaging can be achieved [3]. However, in patients with cardinal features of complicated intestinal obstruction, such as high fever, persistent hypotension and abdominal rigidity, urgent surgical intervention should be sought. We report our experience with an adult male patient who had a congenital trans-mesentric hernia, involving nonviable small and large bowel which both needed resection. It is quite challenging to resect nonadjacent portions of the intestine, as it will lead to difficulty in deciding the configuration of GI re-anastomosis, whether this involved forming a protective stoma or not. However, this work has been reported in line with the SCARE criteria [4].

## 2 Case report

**Fig. 1:**
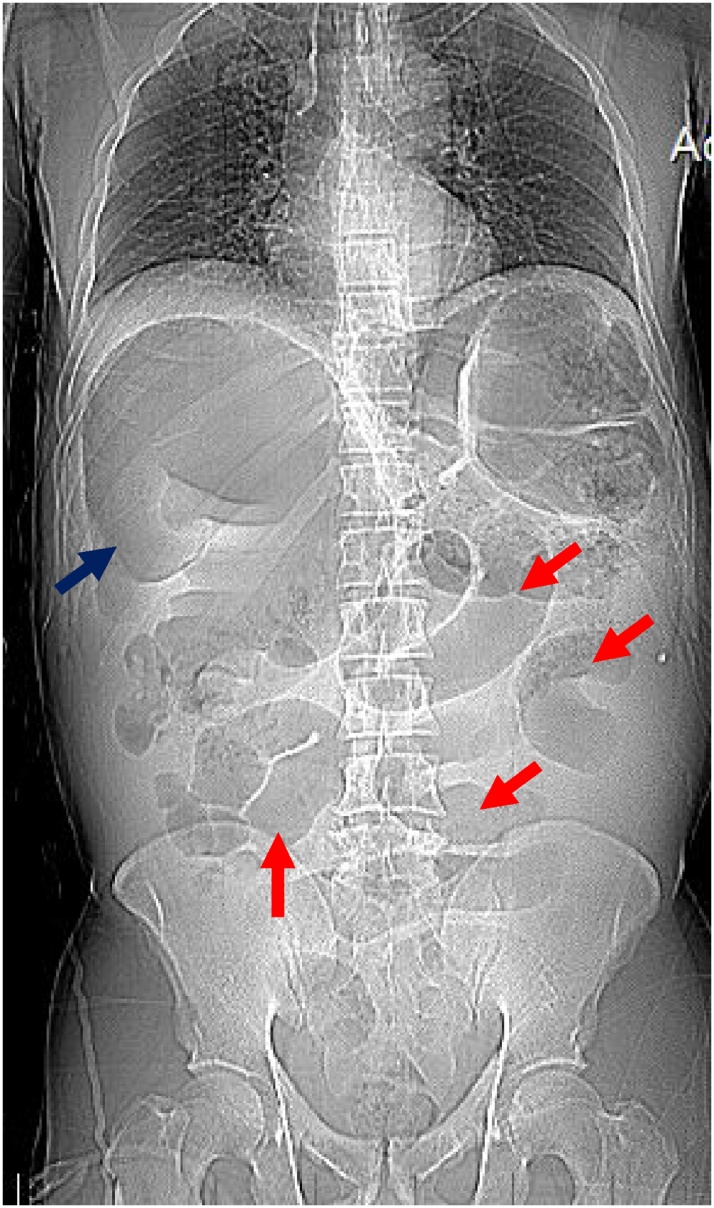
Plain abdominal x-ray in supine position (Red arrows: multiple dilated small bowel loops (jejunum and ileum). Blue arrow: Air fluid level in large bowel loop in the right upper quadrant suspected to be sigmoid colon).

A 47-year old male patient presented to the emergency department of our center with a 4-day history of moderate abdominal pain increasing gradually in severity over the duration of illness. The pain started as generalized all over the abdomen, and then mobilized to the right iliac fossa. The pain was associated with frequented vomiting which started as greenish juice then became food particles. It was also associated with increased an abdominal girth and obstipation. Physical assessment showed a distressed patient with a respiratory rate of 22 and heart rate of 120 bpm. His Blood pressure was 90/60 and Temp 37.8° C orally. O2 Saturation was 90 at room air. Abdominal examination revealed marked abdominal distention, with localized guarding in right iliac fossa. On Digital rectal examination there was no masses or blood and no stool. On presentation, laboratory findings included elevated Creatinine (1.5 mg/dl), BUN (27.0), Na (143) and normal WBC count (5900 cell/mm^3^), hemoglobin (10.4 g/dl), platelets (136 × 10^3^/μl), INR 1.7. Plain abdominal radiographs showed dilated small and large bowel loops (Fig. 1).

**Fig. 2:**
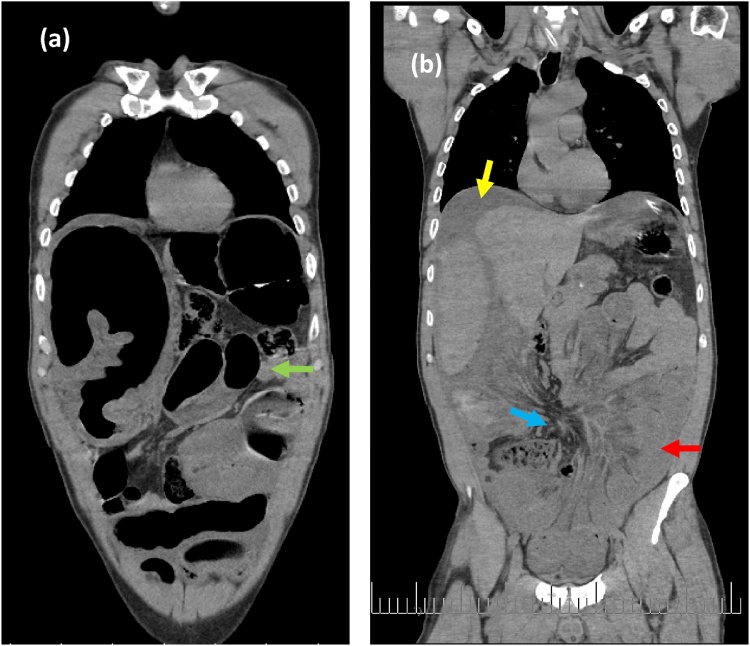
Abdominal CT with contrast, two coronal views: (a) white arrow shows suspected sigmoid volvulus in the right upper quadrant. Green arrow shows dilated small bowel loop. (b) yellow arrow demonstrates preihepatic free fluid, red arrow points at non-enhancing small bowel loop and edematous roots of mesentery, blue arrow shows swirl sign and the suspected site of hernia aperture.

**Fig. 3:**
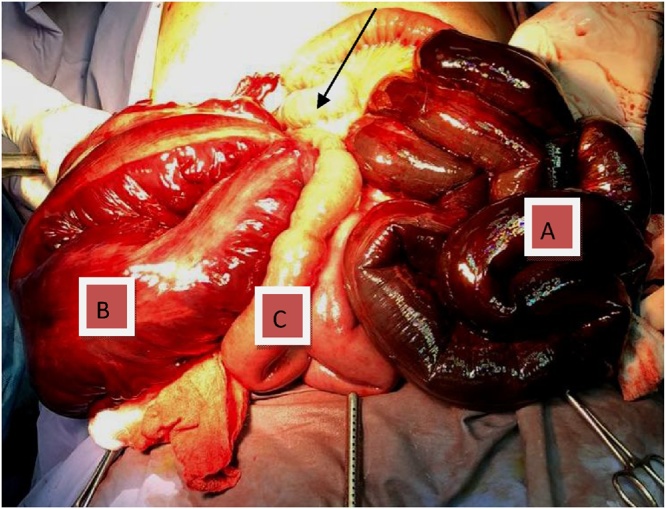
Intraoperative surgical site showing left side of patient (A) Gangrenous distal ileum, right side of patient (B) gangrenous sigmoid colon, (C) viable jejuna loop. The arrow points at the site of trans-mesentric herniation.

The abdomen CT scans with contrast enhancement was advised after resuscitating the patient and stabilizing his condition, and showed dilated small and large bowel with sigmoid volvulus plus large amount of preihepatic and pelvic free fluid. Swirl sign at the mesentery of cecum suspected strangulated bowels due to transmesentric defect (Fig. 2). Intraoperative findings: 2 × 4 cm defect in the mesentery of cecum, Gangrenous sigmoid volvulus and part of the terminal ileum entrapped through the mesenteric gate, and much free fluid (Fig. 3). Surgical treatment started with deflating the sigmoid colon, and reducing it through the defect. We also reduced the small bowel. After that, resection of all nonviable segments was performed. End-to-end anastomosis of small bowel, colostomy on the left side, and closure of defect was done. Immediately after recovery from surgery, the patient was transferred to ICU and stayed for three days, during which he developed atelectasis and systemic inflammatory response with acute kidney injury and blood hemolysis but these rapidly reverted to normal. Following that, the patient was transferred to surgical ward in which he spent seven days with a complication of superficial wound infection and ileus. The infection was treated with IV antibiotics according to antimicrobial susceptibility testing and dressing was applied. Ileus was treated conservatively. On the tenth postoperative day, the patient was discharged with functioning stoma and excellent general condition. We are planning to restore GI continuity after 3 months.

## 3 Discussion

**Fig. 4:**
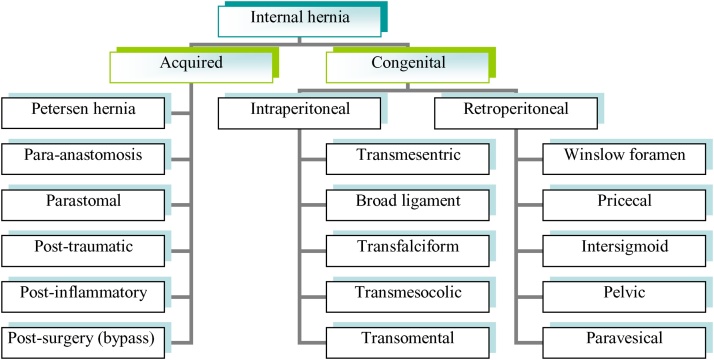
Classification of internal hernia.

The incidence of internal hernia is less than 1% making it a rare malformation of the gastrointestinal tract [5]. Interestingly, congenital transmesentric type accounts for only 8% of all internal hernias. Many hypotheses were proposed to explain the etiology of internal hernia. Treves theory described the weakest area in the mesentery of terminal ileum in 1885 [6]. Ghahremani classified internal hernia into: congenital (including retroperitoneal or retroperitoneal) and acquired [7,8]. Fig. 4 shows two categories of internal hernia, the acquired form is more common in the adult population while congenital type is more common in pediatrics. Some authors suggest that the increasing incidence of acquired internal hernia may be attributed to the raised number of performed hepatobiliary and bariatric bypass surgeries [9]. Small bowel loops have been found to be the most likely herniated content followed by the sigmoid colon. We have found only a minority of published work talking about concomitant small and large bowel internal herniation [10,11]. Due to the wide spectrum of potential presentations of internal hernia, preoperative diagnosis is difficult [12]. Therefore, the clinician must have a high index of suspicion, especially in patients who previously underwent bypass surgeries [13]. Nowadays, Abdominal CT scan with contrast plays the major role in the diagnosis and in planning the surgical management [2,3]. Surgical treatment must address early reduction of the hernia in order to prevent necrosis of the intestine as much as possible [14,15]. Operative treatment should focus on delicate handling of the bowel, reduction of hernia, resection of nonviable segments, searching for other possible silent defects, preventing recurrence and a reasonable method for GI reconstruction. As known, re-anastomosis carries a risk of leak, which is higher in emergency surgery. Creating a stoma has its own merit of complications as well. Surgical options for our patient were: to re-anastomose small bowel and do Hartmann procedure, to restore large bowel continuity and create a double barrel ileostomy, to re-anastomose both large and small bowel with or without protective loop-ileostomy or (finally) to create both ileostomy and colostomy without re-anastomosis. Patient co-morbidities, stability, and surgical experience of anastomosis techniques all played an essential role in determining the appropriate modality of surgical treatment [16]. In our patient's case, the option of re-anastomosing small bowel and Hartmann procedure was the best option since creating double barrel ileostomy predisposes for high output ileostomy. In addition, the distal viable segment of ileum was only 10 cm in length proximal to ileocecal valve. Thus, this carries a higher risk of complication and difficulty with restoration of GI continuity.

## 4 Conclusion

Whether the patient presenting with intestinal obstruction has a history of undergoing previous surgeries (for any reason) or not, the diagnosis of internal hernia must be kept in mind. Clinical sense and early diagnosis when possible aid tremendously in prevention of catastrophic consequences (ischemia, necrosis, perforation and septic shock). Trying to prove the diagnosis of internal hernia radiologically without consideration of patient clinical status may delay surgical intervention thereby increasing mortality and morbidity. Finally, involvement of both Small and large bowels by the obstruction can lead to strangulation and eventually resection need to be performed. This may necessitate colostomy versus ileostomy.

## Please state any conflicts of interest

The authors declare no conflicts of interests.

## Please state any sources of funding for your research

The authors did not receive any fund.

## Ethical approval

This case report is exempted from ethical approval by our institution.

## Consent

The authors state that they have written and signed consent from the patient to publish this report.

## Author contribution

**Dr. Malek Al-Omari:** Conceptualization, Methodology, Validation, Investigation, Writing – original draft, Writing – review and editing, Visualization, Supervision. **Dr. Mohammad Al-Doud**: Formal Analysis, Resources, Data curation, Writing – review and editing.

## Registration of research studies

The authors don’t need to register this work.

## Guarantor

Dr. Malek A. Al-Omari, the corresponding author of this manuscript accept full responsibility for the work and the conduct of the study, access to the data and controlled the decision to publish.

## Provenance and peer review

Not commissioned, externally peer-reviewed.
